# Self-Conjugation of the Enteropathogenic* Escherichia coli* Adherence Factor Plasmid of Four Typical EPEC Isolates

**DOI:** 10.1155/2017/6325736

**Published:** 2017-10-26

**Authors:** Claudia Silva, Crispín Zavala-Alvarado, José L. Puente

**Affiliations:** Departamento de Microbiología Molecular, Instituto de Biotecnología, Universidad Nacional Autónoma de México, Cuernavaca, MOR, Mexico

## Abstract

The enteropathogenic* Escherichia coli* (EPEC) adherence factor plasmid (pEAF) encodes the proteins involved in the biogenesis of the bundle-forming pilus (BFP), a key virulence factor that mediates microcolony formation and the localized adherence phenotype on the surface of the host enterocytes. The presence or absence of this plasmid defines typical EPEC (tEPEC) and atypical EPEC (aEPEC), respectively. Although lateral transfer of pEAF has been evidenced by phylogenetic studies, conjugal transfer ability has been experimentally established only for two pEAF plasmids from strains isolated in the late 60s. In the present work, we tested the self-conjugation ability of four pEAF plasmids from tEPEC strains isolated between 2007 and 2008 from children in Peru and the potential of aEPEC to receive them. A kanamycin resistance cassette was inserted into donor pEAF plasmids in order to provide a selectable marker in the conjugation experiments. Two aEPEC isolated from the same geographic region were used as recipient strains along with the laboratory* E. coli *DH5*α* strain. Here we show that the four pEAF plasmids tested are self-conjugative, with transfer frequencies in the range of 10^−6^ to 10^−9^. Moreover, the generation of aEPEC strains harboring pEAF plasmids provides valuable specimens to further perform functional studies.

## 1. Introduction

*Escherichia coli *is gamma Proteobacteria that colonizes the gastrointestinal tract of humans and other animals [1, 2]. Enteropathogenic* E. coli* (EPEC) is a leading cause of infantile diarrhea in developing countries [3]. EPEC infection is accompanied by a distinct intestinal histopathology, called attaching and effacing (A/E) lesion, characterized by the intimate adherence of bacteria to enterocytes, the formation of actin-rich pedestals underneath the sites of bacterial attachment, and localized destruction of the brush border microvilli [4]. The genes necessary for the establishment of the A/E lesion are located within the pathogenicity island known as the locus of enterocyte effacement (LEE) [5].

EPEC is classified into typical (tEPEC) and atypical (aEPEC) strains based on the presence of a large virulence plasmid known as EPEC adherence factor plasmid (pEAF) [1, 6, 7]. The pEAF encodes the bundle-forming pilus (BFP) that mediates EPEC autoaggregation and microcolony formation on the surface of epithelial cells, a phenotype known as localized adherence [8–11], as well as dispersion of the bacteria through the intestinal mucosa and virulence in adult volunteers [12–14]. BFP biogenesis is specified by the 14-gene* bfp *operon [15, 16].

Although the pEAF is not essential for the formation of the A/E lesion [17], it enhances their efficiency by promoting localized adherence on the host cell and the expression of LEE genes through the* per* regulatory operon, which consists of three genes,* perA, perB*, and* perC*. PerA activates the expression of the* bfp* operon and autoactivates its own expression [18–22]. PerC increases the expression of LEE encoded proteins by enhancing the activation of the LEE-encoded regulator (Ler) [20, 23–25].

The pEAF from prototype strains B171 (O111:NM), isolated in Washington in 1983 [26], and E2348/69 (O127:H6), isolated in Tauton, UK, in 1969 [27], has been fully sequenced. The most conspicuous difference between these two pEAF plasmids is the presence of conjugal transfer* (tra)* genes on E2348/69 pMAR7, a derivative of the wild-type pMAR2 marked with a Tn801 transposon conferring resistance to ampicillin [8, 28], but not in the B171 pEAF, pB171 [29]. However, the rest of the two plasmids are highly conserved, and they share the genetic organization of* bfp* and* per* operons, and three plasmid replication and maintenance regions (repFIIA, repFIB, and* stb*-*par*-*rsv*-*ccd*) [28, 29]. The presence of* tra* genes in other pEAF has been determined by Southern-blot hybridization [28] and genome sequence analysis of tEPEC strains of different phylogenomic lineages [30], but their conjugation ability was not tested experimentally. To our knowledge the E2348/69 pMAR7 and E2347/69 pDEP1, both strains isolated from the same outbreak and belonging to serotype O127:H6, are the only pEAF plasmids for which conjugative transfer ability has been demonstrated [8, 31, 32].

The aim of this work was to test the self-conjugation ability of four EAF plasmids from tEPEC strains isolated between 2007 and 2008 from children in Peru [33] and the potential of two aEPEC strains isolated from the same geographic region to incorporate them when used as recipient strains in conjugation experiments.

## 2. Materials and Methods

### 2.1. EPEC Strains

The EPEC strains used in this work were isolated in 2007-2008, as part of an epidemiological surveillance program in suburban areas of Lima, Peru [33]. Based on the presence of EAF plasmid markers (see below) four tEPEC strains were selected as pEAF plasmid donors, and two aEPEC strains as recipients.* E. coli *DH5*α* was also included as a recipient strain (Table 1).

### 2.2. PCR Detection of pEAF Genes

Based on available pEAF sequences, primers specific for the amplification of the repFIIA replication region and of two distant regions within the* tra* operon (*traI* and* traC* genes, encoding for the relaxase, and an ATPase required for pilus production of the type IV secretion system, respectively), were designed using the primers4clades web server [34] (Table 2). For the detection of the* bfp* operon, the* bfpA* sequence was amplified using the primers described by Lacher et al. [35]. DNA was extracted from liquid cultures by a modification of the salt extraction method described by Miller et al. (1988) [36]. Amplifications were performed in 50 *μ*l reactions using a commercial Taq polymerase kit (Thermo scientific) and 1.5 U Taq polymerase per tube, with a final concentration of 1.5 mM MgCl, 0.2 mM dNTPs, and 0.5 *μ*M each primer. Two *μ*L of extracted total DNA was used as a template (roughly 50 ng). The cycling program was as follows: 5 min 95°C followed by 30 cycles of 45 s at 94°C, 30 s at 57°C, and 45 s at 72°C and completed by a final extension for 5 minutes at 72°C.

### 2.3. Insertion of a Kanamycin Resistance Cassette into pEAF

We used the Lambda Red Recombinase system [38] to insert the kanamycin resistance cassette (Km^R^) carried in pKD4 into the pEAF ISS*fl1* locus of the four selected strains (Table 1) using the recombinase function carried on pKD78. The ISS*fl1* locus was selected since this was the target for the insertion of the ampicillin resistance Tn*801* transposon in EPEC E2348/69 pMAR7 [8]. The K1 and K2 primers in combination with the IS-F and IS-R primers were used to confirm the correct insertion of the kanamycin resistance cassette into ISS*fl1* (Table 2).

### 2.4. Conjugation Experiments

The strains carrying the pEAF-derivatives marked with the kanamycin resistance cassette were used as donors in mating experiments (Table 1).* E. coli* DH5*α* and two Peruvian aEPEC strains (D3319 and D3264) were used as recipients. Strains D3264 and DH5*α* were Nal^R^, and a spontaneous Nal^R^ colony was selected for D3319. In order to avoid the selection of spontaneous Nal^R^ donor strains instead of transconjugants during the conjugation experiments, spontaneous rifampicin resistant (Rif^R^) colonies of the donor strains were selected to have a second antibiotic resistance selection marker. Therefore, the Rif^R^ and Nal^R^ recipient strains were named DH5*α*RN, D3319RN and D3264RN (Table 1). Conjugation experiments were performed as follows: 5 ml LB overnight liquid cultures of donor and recipient strains were pelleted, washed and resuspended in 1 ml of sterile water, mixed in a 1 : 10 donor-to-recipient ratio (50 : 500 *μ*l), platted onto nonselective solid LB plates, and incubated overnight at 37°C. The conjugation mix was removed from the LB plate with 1 ml of sterile water, and transconjugants were selected by plating 100 *μ*l of serial dilutions onto solid LB medium supplemented with kanamycin (60 *μ*g/ml) to evaluate the number of donors, or rifampicin (100 *μ*g/ml), nalidixic acid (30 *μ*g/ml), and kanamycin (60 *μ*g/ml) to quantify the number of transconjugants. Conjugation frequencies were calculated as the ratio of number of transconjugants (Km^R^, Rif^R^, Nal^R^)/number of donor strains (Km^R^). Each conjugation experiment was repeated at least twice. The presence of the pEAF plasmid was confirmed by* bfpA *PCR analysis of ten transconjugant colonies per experiment (Table 2).

**Figure 1:**
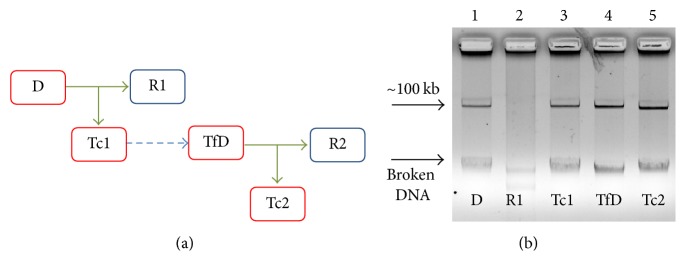
Schematic and plasmid profile representation of the first- and second-generation conjugation experiments. (a) A donor (D) tEPEC/pEAF::*km* strain was challenged for conjugation with recipient (R1) Rif^R^ and Nal^R^ aEPEC or* E. coli *DH5*α* (Table 1). Subsequently, the pEAF::*km* plasmid from a transconjugant colony (Tc1) was purified and transformed by electroporation into* E. coli *DH5*α*. The transformant (TfD) DH5*α*/pEAF::*km* was used as donor for conjugation with recipient strain DH5*α*RN (R2), from which transconjugant colonies (Tc2) were derived. (b) Plasmid preparations of donor (D) strain D0131/pD0131::*km* (1), recipient (R1) strain D3264RN (2), transconjugant (Tc1) strain D3264RN/pD0131::*km*, obtained from the first-generation conjugation (3), transformant (TfD) DH5*α* harboring the transconjugant pD0131::*km*, used as donor in the second-generation conjugation experiment (4), and transconjugant strain D3264RN harboring the pD0131::*km* (Tc2), obtained in the second-generation conjugation experiment (5).

To corroborate the self-conjugation ability of the pEAF plasmids, the purified plasmid from a transconjugant colony derived from the first conjugation event was transformed by electroporation into DH5*α* using kanamycin as selection marker. Transformant colonies were checked by PCR amplification of* bfpA*. A transformant DH5*α* strain harboring the pEAF was used as donor in further conjugation experiments using DH5*α*RN as the recipient strain (Figure 1(a)).

### 2.5. Plasmid Profiling

To analyze the plasmid content of selected isolates, a modified protocol of the alkaline lysis procedure proposed by Kieser was used [39]. The products were separated in 0.7% agarose gels in 1x TBE buffer at 100 volts for 4 hours, stained with a 1% ethidium bromide solution, and photographed.

## 3. Results

### 3.1. tEPEC Strains D0131, D3152, D3048, and D3129 Contain pEAF and Conjugative Transfer Markers

EPEC isolates D0131, D3152, D3048, and D3129, previously reported as tEPEC [33] (Table 1), were selected to analyze the conjugative transfer ability of their pEAF plasmids, which were positive for* bfpA*,* repFIIA*,* traI*, and* traC* genes based on PCR amplification using specific primers (data not shown). This observation suggested that these plasmids are potentially conjugative. aEPEC strains D3319 and D3264 were selected as recipient strains (Table 1).

### 3.2. The pEAF Plasmids Were Successfully Marked with a Kanamycin Resistance Cassette

In order to assess the conjugation ability of the pEAF plasmids of tEPEC strains D0131, D3152, D3048, and D3129, these were marked with a kanamycin resistance cassette, since the* E. coli* virulence plasmids rarely carry antibiotic resistance genes [40, 41]. The region selected for its insertion was the IS element ISS*fl1, *located downstream of the* per* operon, because it was the target sequence for the Tn*801* transposon inserted in EPEC E2348/69 derivative pMAR7, which was demonstrated not to affect the pEAF adherence functions [8]. The pEAF plasmids of these strains were successfully marked and their derivatives were referred to as pD0131::*km*, pD3152::*km*, pD3048::*km*, and pD3129::*km*, respectively (Table 1).

For the conjugation experiments aEPEC recipient strains D3319 and D3264 were chosen, as well as the* E. coli* laboratory strain DH5*α*. For the three recipient strains spontaneous Rif^R^ colonies were selected to obtain a second resistance marker to counter-select the sensitive donor strains. These Rif^R^ and Nal^R^ recipient strains were referred to as D3319RN, D3264RN, and DH5*α*RN (Table 2).

### 3.3. The pEAF Plasmids Are Self-Conjugative

The conjugation experiments showed, in agreement with the presence of* tra *genes, that all four tEPEC strains successfully transferred their pEAF plasmids to the three recipient strains. The transfer frequencies were low in the range between 10^−6^ and 10^−9^ (Table 3). No consistent differences in transfer frequencies among recipient strains were observed. Our results demonstrate that pEAF plasmids from different tEPEC strains, other than the prototype, are able to self-conjugate to other* E. coli* strains.

In order to confirm the self-conjugation ability of the pEAF plasmids, the transconjugant plasmids from mating experiments between strain D0131 pD0131::*km* and D3264RN, and strain D3129 pD3129::*km* and D3319RN, were transformed into DH5*α*. The DH5*α* transformant strains carrying pD0131::*km* or pD3129::*km* were used as donors in a second conjugation experiment with DH5*α*RN as the recipient strain (Figure 1(a)). The conjugation frequencies for DH5*α* pD0131::*km* were in the range of 1.5 × 10^−6^ to 6.7 × 10^−8^ and for DH5*α* pD3129::*km* in the range of 1.1 to 5.1 × 10^−6^. Transconjugant colonies were positive for* bfpA* amplification. Plasmid profiles for the conjugation experiments of strain D0131 showed that a plasmid of about 100 kb was transferred to both D3319RN and DH5*α*RN (Figure 1(b)). Of note, purified pD0131::*km* from the second-generation D3319RN transconjugant (Figure 1(b), lane 5) looks slightly shorter than that from the first-generation D3319RN transconjugant (Figure 1(b), lane 3), which could be indicative of loss of genetic material during the conjugation process. However, taken together, these results demonstrate that the pEAF plasmids contain the complete molecular machinery for self-conjugation irrespective of the* E. coli* donor genetic background.

## 4. Discussion

In this study, we report that the pEAF plasmids from four tEPEC strains isolated in 2007-2008 from children in Peru are self-conjugative. To our knowledge, self-conjugation ability has been reported only for the highly similar pMAR7 and pDEP1 pEAF plasmids from two EPEC O127:H6 isolates [31, 32]. For tEPEC strain B171 (O111:NM), Riley et al. (1987) initially reported that it was able to transfer localized adherence and antibiotic resistance phenotypes [42]; however, sequence studies showed that the B171 pEAF does not carry* tra* genes [29], and later Nwaneshiudu et al. (2007) demonstrated that the transfer of the adherent and antibiotic resistance phenotypes was due to the mobilization of B171 pEAF by a conjugative antimicrobial resistance plasmid [43]. The pEAF of our strains were* tra* positive, and subsequent mating experiments supported their self-conjugation (Table 3 and Figure 1).

Our experiments also demonstrate that aEPEC strains can receive the pEAF. These transconjugant strains are suitable candidates for studying the effect of the introduction of the pEAF into aEPEC genetic backgrounds, as well as its stability under laboratory conditions considering that pEAF instability has previously been reported [30, 44, 45]. Future research will also be conducted to analyze the functional responses and gene expression profiles of these aEPEC pEAF-carrying strains, including the expression of BFP and the localized adherence phenotype, as well as the regulatory effect of the* per* locus over the LEE island. In this regard, recent global transcriptional analyses have demonstrated that pEAF genes influence the expression of a number of chromosomal genes in addition to the LEE [30, 46].

Studies tracing the evolution of EPEC have provided phylogenetic evidence for the multiple gain and loss of the pEAF virulence plasmids [30, 47–49]. Although our mating experiments show low conjugation frequencies (10^−6^ to 10^−9^), the present study provides experimental evidence for the self-conjugation of pEAF virulence plasmids between* E. coli* strains, supporting the role of pEAF transfer in EPEC ecology and evolution.

## 5. Conclusions

In this study four tEPEC strains were challenged for conjugation of their pEAF virulence plasmids. All the pEAF plasmids were able to self-conjugate into three* E. coli *recipient strains including a laboratory strain, with transfer frequencies ranging from of 10^−6^ to 10^−9^. In a second-generation experiment, two of the transconjugant pEAF plasmids were able to conjugate even from a non-EPEC genetic background (DH5*α*), indicating that these plasmids fully possess the ability for lateral transfer. Moreover, the generation of transconjugant aEPEC strains harboring pEAF plasmids provides valuable specimens to further perform functional response analysis.

## Figures and Tables

**Table 1:** Strains used in this study.

Strain	Features	Source
*tEPEC*		
D0131	Wild-type tEPEC strain	[33]
D3152	Wild-type tEPEC strain	[33]
D3048	Wild-type tEPEC strain	[33]
D3129	Wild-type tEPEC strain	[33]
*aEPEC*		
D3319	Wild-type aEPEC strain	[33]
D3264	Wild-type aEPEC strain, Nal^R^	[33]
*Laboratory*		
DH5*α*	Laboratory strain, Nal^R^	[37]
*Donors*		
D0131 pD0131::*km*	D0131/pEAF::*km* derivative	This study
D3152 pD3152::*km*	D3152/pEAF::*km* derivative	This study
D3048 pD3048::*km*	D3048/pEAF::*km* derivative	This study
D3129 pD3129::*km*	D3129/pEAF::*km* derivative	This study
*Recipients*		
D3319RN	D3319 spontaneous Nal^R^ and Rif^R^ derivative	This study
D3264RN	D3264 spontaneous Rif^R^ derivative	This study
DH5*α*RN	DH5*α* spontaneous Rif^R^ derivative	This study

**Table 2:** Primers used in this study.

Primer name	Region	Sequence 5′ to 3′	Reference
*pEAF *			
bfpA_114F	*bfpA*	GTCTGCGTGTGATTCCAATA	[35]
bfpA_521R		TCAGCAGGAGTAATAGC	[35]
FIIA-F	repFIIA	CCTTCACACGACGTTCCACT	This study
FIIA-R		CGCCAGGTAAAGAACCCGAA	This study
traI-F	*traI*	GAGCTGGGTAAAGAGCAGGTCATGG	This study
traI-R		CAGGTTTGTTCTCTGCCATTTT	This study
traC-F	*traC*	GTCGGGRAGATGATTAACCATAA	This study
traC-R		ACCRACYTTRYGATTTTTGAAGTC	This study
ISSfl1-F	ISS*fl1*	TCGCCTCATGGGTAATGGGG	This study
ISSfl1-R		AATGGCGAGTGTCGAAGAACA	This study
*Mutagenesis*			
ISSf11-H1	ISS*fl1*	CATGTCCTTCGTGCCAGCCTTCTGTGTGACGGGCGTTCCAT	This study
		TGTAGGCTGGAGCTGCTTCG	
ISSf11-H2		ACCGAGTAACCACATTACGATAGTGCTCAACGTTGCCAGCAG	This study
		CATATGAATATCCTCCTTAG	
K1	*kan*	CAGTCATAGCCGAATAGCCT	[38]
K2		CGGCCACAGTCGATGAATCC	[38]

**Table 3:** Frequencies of conjugal transfer of the pEAF plasmid of four tEPEC strains.

	Recipient strains^b^		
Donor tEPEC strain	DH5*α*RN	D3319RN	D3264RN
D0131 pD0131::*km*	1.1 × 10^−7^ to 7.9 × 10^−8^	2.8 to 4.2 × 10^−8^	1.3 × 10^−8^ to 7.5 × 10^−8^
D3152 pD3152::*km*	2.8 × 10^−6^ to 2.5 × 10^−8^	2.3 × 10^−7^	8.2 × 10^−8^ to 9.4 × 10^−8^
D3048 pD3048::*km*	5.2 × 10^−7^ to 9.3 × 10^−7^	1.8 × 10^−6^ to 3.6 × 10^−9^	1.0 × 10^−7^ to 6.2 × 10^−9^
D3129 pD3129::*km*	2.4 × 10^−6^ to 2.0 × 10^−8^	1.5 × 10^−6^ to 3.3 × 10^−7^	1.3 × 10^−7^ to 5.6 × 10^−8^

The experiments were repeated at least twice. ^b^Rifampicin and nalidixic acid resistant derivatives.
